# Ophthalmology and Otology

**Published:** 1896-05

**Authors:** Alvin A. Hubbell

**Affiliations:** Buffalo, N. Y., Professor of ophthalmology and otology in the Medical Department of Niagara University


### Progress in Medical Science.

### OPHTHALMOLOGY AND OTOLOGY.

CONDUCTED BY ALVIN A. HUBBELL, M. D., Buffalo, N. Y.,
Professor of ophthalmology and otology in the Medical Department of Niagara University.

### HYDROZONE IN PURULENT OTITIS MEDIA.

BOTELER, Kansas City, (*Medical Bulletin*, February, 1896,) reports a case in which the patient, aged 24 years, laborer, complained for about four weeks of intense pain in his left ear, so severe at times that he apparently lost consciousness. The man's face was deformed ; an edematous swelling, the size of a baker's half-loaf, occupied the usual location of the ear ; auricle almost buried in edematous tissue ; intensely tender ; integument and subcutaneous tissues were thoroughly infiltrated ; ichorous, fetid pus exuded from an almost imperceptible meatus ; patient chilly and showed signs of septic infection ; indications pointed to suppuration of the mastoid cells. Treatment consisted of heroic doses of elixir of the six-iodides internally ; small Eustachian catheter was introduced into the external meatus, rubber syringe attached and through this half an ounce of hydrozone was injected four times a day. Patient was confined in bed and hot fomentations applied. In twenty-four hours the intensity of the odor, amount of the discharge and size of the swelling were materially reduced. Hydrozone then was injected through a larger catheter every hour, which was continued for a week. At the end of eight days the swelling had entirely disappeared, when an examination disclosed a circular perforation of the ear drum, proving the case to have been one of purulent otitis media with systemic septic infection. Two incisions were made to permit the escape of pus from the integument ; the mastoid was not involved. The rapidity with which the disease yielded after the introduction of hydrozone seemed remarkable.

---

### TREATMENT OF GRANULAR OPHTHALMIA AND SOME OTHER AFFECTIONS BY APPLICATION OF IODINE IN LIQUID VASELINE.

DR. E. A. NEZNAMOW, privat-docent of ophthalmology at the medical faculty of Kharkow, has employed a new method of treatment in trachoma, from which remarkably good results appear to be obtained. This treatment consists in applying twice a day to the mucous membrane of the everted eyelid a solution of pure iodine in liquid vaseline. The applications are made with a brush or small cotton swab steeped in the solution, the strength of which varies according to the particular form of the affection in each case. In the chronic, cicatricial varieties of granular ophthalmia, complicated by pannus, infiltration, ulceration and superficial opacities of the cornea, Dr. Neznamow employs liquid vaseline containing from ½ to 1 per cent. of iodine. By this means marked improvement is obtained within three or four days ; in two or three weeks the vessels of the pannus become obliterated, the exudations are absorbed, the cornea recovers its transparency, the palpebral mucosa becomes smooth and soft, and as a result thereof sight is improved.

The so-called pannus cornosus or crossus is said to yield with surprising rapidity to applications of liquid vaseline containing 1.5 per cent of iodine. In the case, for instance, of a patient in whom two-thirds of the cornea were covered by a pannus, at least half a millimeter in thickness, the left eye, after three weeks' treatment, presented no abnormal symptoms, except slight superficial opacity of the cornea, while the pannus, which remained in the right eye, was insignificant.

In recent granular and papillary pannus it is necessary, in order to obtain the desired result, to increase the quantity of iodine in the solution to 3 or even 5 per cent. Liquid vaseline, however, does not dissolve more than 1.5 per cent. of this substance, and it must therefore be mixed with a little sulphuric ether, or, better still, rectified petroleum, in sufficient quantity to insure the required concentration of the solution.

Applications of these strong solutions to the palpebral conjunctiva, as a rule, determine rather intense phenomena of reaction. The mucosa assumes a red tint, more or less profuse lachrymation supervenes, and the patient experiences a smarting pain, which, however, speedily subsides. After four or five applications, a catarrhal state is induced, with copious secretion, congestion and a slight swelling of the mucosa. At this stage of the treatment, Dr. Naznamow, while continuing the iodine applications two or three times daily, slightly cauterises the conjunctiva now and then with a 2 per cent. silver nitrate solution, followed immediately by irrigation of the eye. In addition, the largest granulations are incised, and their contents squeezed out. By these means the granulation becomes absorbed within a fortnight or three weeks.

In cases of recent trachoma, in which the secretion is profuse from the onset, it is advisable, before resorting to the use of strong iodine solutions, to make a few applications of glycerine containing 5 per cent of iodine, in order to check the hypersecretion of the palpebral conjunctiva.

The excellent effects of the treatment introduced by Dr. Neznamow have been confirmed by Dr. L. L. Hirschmann, professor of ophthalmology at the faculty of Kharkow, who has found that iodised liquid vaseline may be employed with advantage, not only in granular ophthalmia, but also in certain other ocular affections. Thus old-standing ciliary blepharitis is said to rapidly improve under application to the edge of the eyelids of a ½ or 1 per cent. solution of iodine in liquid vaseline.

Instillation into the conjunctival cul-de-sac of a few drops of the same solution are also said to give very good results in cases of dacryo-cystitis.

Lastly, persistent infiltration following parenchymatous peratitis is said to be rapidly absorbed under the influence of applications to the palpebral conjunctiva of 2, 3 or 5 per cent. iodised vaseline. Solutions of iodine in liquid vaseline, obtained by the addition of sulphuric ether or petroleum, should be kept in tinted, well-stoppered vials, so as to exclude air and light. They remain clear for about a week, after which they become cloudy and unfit for use.—*The Medical Week*, Dec. 27, 1895.

---

### THE TREATMENT OF WOUNDS OF THE CORNEA BY CONJUNCTIVAL OCCLUSION.

Dr. de Wecker, Paris, says the employment of a conjunctival flap in wounds or incisions of the cornea and sclera, as advocated by Snellen, is easily applicable when the wound is in the sclera at some little distance from the cornea; less available when the wound is close to or at the corneal margin. In cases in which the wound is in the central part of the cornea, the difficulty of covering and keeping it covered by a conjunctival flap is considerable. Even if two flaps be made on opposite sides of the cornea and joined medially by sutures, the traction is sufficient to make the stitches cut their way out before the conjunctiva can have become united to the wound or the wound have healed beneath the con junctiva.

The writer thinks he has found a means of coping successfully with this difficulty by making, temporarily, a complete conjunctival covering to the cornea. He employs this proceeding only in cases of large wounds reaching to or extending beyond the corneal limbus and showing a tendency to gape more or less widely. The method recommended is as follows: The conjunctiva and subconjunctival tissue are carefully divided all around and close to the margin of the cornea. The conjunctiva is then separated from the deeper tissues almost as far as the insertion of the recti muscles, and when thus freed from its attachments is drawn over the cornea and stitched, either by a continuous suture, which brings the cut margins together, like the drawing-string at the mouth of a bag, or by four or six separate sutures. Care should be taken that the stitches are passed through the subconjunctival tissue, in order that they may not cut out prematurely. The cornea is thus covered in its entirety by conjunctiva. The edges of the eyelids and the cilia having been carefully cleansed, a dressing and bandage are applied and allowed to remain undisturbed for eight or ten days, until the suture threads have become spontaneously detached.

In the author's experience there has been no case in which the adhesion of the conjunctiva to the cornea was more extensive than was desirable. It has always left unaffected the part of the cornea which had not been wounded, its adhesion to the cornea being limited to a narrow area adjoining the lips of the wound.

By this treatment de Wecker maintains that it is possible to save eyes which have sustained very large wounds of the cornea, if the operation is performed (under general anesthesia) very soon after the accident; time should not be taken to deal with deeper lesions, such as wound or dislocation of the lens, which can be better treated after the subconjunctival healing of the corneal wound has taken place.

The operation, the writer adds, is very easily performed and leaves no visible scar.—*Annales d' Oculistique*, November, 1894, and *Ophthalmic Review*, February, 1895.

---

### THE OPERATIVE TREATMENT OF IMMATURE, AND SOME FORMS OF ZONULAR, CATARACT.

Dr. John E. Weeks, of New York, at a meeting of the American Medical Association, 1895, read a paper on the operative treatment of immature, and some forms of zonular, cataract, in which he stated that the most favorable time for the removal of cataract was at the period when the lens fibers had been separated fro m the lens capsule, by the formation of liquor Morgagni between them. A stage of swelling was spoken of as existing in the formation of soft and of rapidly-developing senile cataract, but was said not to exist in the cases of nuclear cataract and of sclerosed lens. Swollen lens fibers were not found to be easily detachable from the lens capsule. In many cases in the formation of ordinary senile cataract, vision may become reduced to 20-100 or less, at which point it may remain for a long period of time. This stage is distressing to the individual, as it renders him unable to perform the ordinary duties of life, and may entail hardships on the patient or patient's family.

The author's experience in the removal of noncataractous lenses at the dead-house led him to believe that all cataractous lenses occurring in individuals twenty-five or more years of age, could be removed with comparative safety as soon as the opacity had progressed sufficiently far to interfere with useful vision.

Förster's operation for the ripening of immature cataract was tried a number of times, but was abandoned, as it was found that the lens cortex, although opaque, was still somewhat adherent to the capsule, and the removal of the cataract, after Förster's operation, was attended with as much difficulty as was experienced in removal without this operation. It also carried with it the chances of the inflammation and bad results incident to the additional operation.

The operation attempted by the author of the paper, in all uncomplicated cases, was simple extraction. As much cortical substance as possible was removed by external manipulation.

Lavage was seldom resorted to. If a small amount of lens cortex remained in the eye, it becomes absorbed and produces no inflammatory trouble. The author has never seen inflammatory conditions awakened by the presence of cortical substance in the eye, after removal of immature cataract.

Twenty-five cases of extraction were reported, divided as follows : Immature, soft cataract, 3 ; zonular cataract, 5 ; immature, complicated cataract, 4 ; immature, diabetic cataract, 1 ; immature, senile uncomplicated, 12. There were no losses. Iridectomy was done in six cases, simple extraction in 19 cases.

There was one prolapse of iris. Dissection of secondary cataract was done in 20 cases. The ultimate results were : 20-20, or better, 13 cases ; 20-30, 2 ; 20-40, 3 ; 20-50, 2 ; 27-70, 5.

The results appeared to the author to be as favorable as those obtained by the removal of cataract at the stage of maturity and the relief to the patients by quick restoration of vision was very great. Such operations should be done by expert operators only.—*The Ophthalmic Record*, July, 1895.

